# Manganese/Yttrium Codoped Strontium Nanohexaferrites: Evaluation of Magnetic Susceptibility and Mossbauer Spectra

**DOI:** 10.3390/nano9010024

**Published:** 2018-12-25

**Authors:** Munirah Abdullah Almessiere, Yassine Slimani, Hakan Güngüneş, Abdulhadi Baykal, S.V. Trukhanov, A.V. Trukhanov

**Affiliations:** 1 Department of Physics, College of Science, Imam Abdulrahman Bin Faisal University, P.O. Box 1982, Dammam 31441, Saudi Arabia; 2 Department of Nano-Medicine Research, Institute for Research & Medical Consultations (IRMC), Imam Abdulrahman Bin Faisal University, P.O. Box 1982, Dammam 31441, Saudi Arabia; abaykal@iau.edu.sa; 3 Department of Biophysics, Institute for Research & Medical Consultations (IRMC), Imam Abdulrahman Bin Faisal University, P.O. Box 1982, Dammam 31441, Saudi Arabia; yaslimani@iau.edu.sa; 4 Department of Physics, Hitit University, Çevre Yolu Bulvarı-Çorum 19030, Turkey; Gungunes@gmail.com; 5 Scientific-Practical Materials Research Centre NAS of Belarus, 19 P. Brovki Street, 220072 Minsk, Belarus; Trukhanov@gmail.com (S.V.T.); truhanov86@mail.ru (A.V.T.); 6 Department of Electronic Materials Technology, National University of Science and Technology MISiS, Leninsky Prospekt, 4, Moscow 119049, Russia; 7 Laboratory of Crystal Growth, South Ural State University, Lenin Prospect, 76, Chelyabinsk 454080, Russia

**Keywords:** MYSNHFs, dopants, Mössbauer spectra, magnetic susceptibility, hyperfine interactions

## Abstract

Manganese (Mn)- and yttrium (Y)-substituted Sr-nanohexaferrites (MYSNHFs) of composition Sr_1−x_Mn_x_Fe_12−x_Y_x_O_19_ (with 0.0 ≤ x ≤ 0.5) were prepared by citrate sol-gel autocombustion method. As-prepared MYSNHFs were characterized via diverse analytical techniques to determine the influence of Mn and Y cosubstitution on their microstructures and magnetic properties. ^57^Fe Mössbauer spectra of the MYSNHFs were used to evaluate the variation in the line width, isomer shift, quadrupole splitting, and hyperfine magnetic field values. It was shown that the dopant ions could preferentially occupy the 12k, 4f_2_, and 2b sites. Furthermore, the observed shift in the blocking temperatures of the studied MYSNHFs towards lower values with rising Mn^2+^ and Y^3+^ contents was attributed to the overall particles size reduction. Meanwhile, the AC susceptibility of the proposed MYSNHFs revealed that the magnetic interactions were weakened with the increase in dopant contents which was ascribed to the replacement of both Sr^2+^ and Fe^3+^ ions by the Mn^2+^ and Y^3+^ dopants.

## 1. Introduction

Nanotechnology science has created great excitement and expectations in the last few years. By its very nature, the subject is of immense academic interest, having to do with very tiny objects in the nanometer regime. There has already been much progress in the synthesis, assembly, and fabrication of nanomaterials, and, of equal importance, in the potential applications of these materials in a wide variety of technologies [1,2,3]. 

In recent times, strontium (Sr) hexaferrites (hereafter named as Sr–HFs) have been intensively studied due to their effectiveness towards microwave (MW) absorption, magnetic recording media, signal processing, telecommunication, MW filtering, audio systems, magneto-optic media, and so forth [4,5,6,7,8]. However, the practical application of such Sr-nanohexaferrites (Sr-nHFs) is strictly affected by the synthesis techniques and preferential site occupation by the dopant ions among the five different Fe^3+^ sublattices such as tetrahedral (4f_1_), trigonal bipyramidal (2b), and octahedral (12k, 2a and 4f_2_) in the hexagonal structure [9]. Meanwhile, Sr–HF systems appear unique wherein their structures allow the favorable substitution of all Fe^3+^ ions by trivalent ions without secondary phase formation [10]. This in turn leads to the procession of varied magnetic traits depending on the nature of dopants’ including magnetic, nonmagnetic and rare-earths and their contents [11]. 

Over the last decades, various strategies have been developed to modify the magnetic and electric properties of Sr–HFs via the partial substitution of Fe^3+^‏ or Sr^2+‏^ ions. W.M.S. Silva et al. [12] investigated the Mn substitution on the structure and magnetic properties of SrFe_12_O_19_ nanoparticles prepared by sol-gel method. They showed that the crystal lattice constants did not change significantly with Mn substitution. Room temperature Mössbauer investigations indicated that Mn ions preferentially occupied the 12k, 4f_1_, 4f_2_, and 2a sites. On the other hand, yttrium (Y)-substituted SrFe_12_O_19_ hexaferrites were prepared through a solid-state reaction technique by S. Jiang et al. [13]. It is found that the single magnetoplumite phase structure transformed into a multiphase structure with the increase of Y content, where a small amount of hematite phase existed in M-type phase. The magnetization measurements showed that the saturation magnetization (M_s_) first increases and then decreases with the increasing of Y content. However, the value of coercivity (H_c_) increases with the increasing of Y content. X.F. Niu and coworkers [14] investigated the structural and magnetic properties of Y-doped Sr–HF. The obtained results revealed that the lattice constant ‘a’ increased first of all and then decreased and ‘c’ increases slowly with increasing Y content. Magnetization investigations indicated that H_c_ and maximum energy product (BH_max_) are first increased and then decreased. Also, D. Shekhawat and P.K. Roy [15] reported the influence of Y substitution on the structural, dielectric, and magnetic properties of Sr–HFs synthesized by the autocombustion approach. The structural analysis indicates that the Y ions reorganize themselves without troubling the parent lattice. M_s_ and M_r_ magnetizations are decreased, however H_c_ and Curie temperature (T_c_) are improved with increasing Y content. The optimized value of BH_max_ was obtained for Sr–HFs substituted with Y.

In recent years, several studies tried to greatly improve the properties of M-type Sr–HFs via the cosubstitution of Ce–Y [16], Nd–Zn [17], La–Co [18], La–Cu [19], Zr–Mn [20], Nd–Co [21], La–Zn [22], Nd–Zn [23], Gd–Sn [24], Pr–Ni [25], Bi–Cr [26], Co–Zr [27], Co–W [28], and Mn–Zn [29]. However, the role of both Mn and Y cosubstitution on the various properties of M-type Sr–HFs has not yet been studied. Accordingly, we studied in the present work the effect of Mn and Y cosubstitution on the structural, morphological, microstructural, and magnetic properties (Mössbauer spectra, AC susceptibility, Magnetization versus applied field) of Sr-nHFs was investigated deeply. So, a series of Sr_1−x_Mn_x_Fe_12−y_Y_y_O_19_ (with varying x = y), where Sr^2+^ and Fe^3+^ ions were partially cosubstituted via Mn^2+^ of and Y^3+^ cations, was prepared.

## 2. Experimental

Analytical grade chemical reagents (purity 99.99%, Sigma-Aldrich, St. Louis, MO, USA) of strontium nitrate [Sr(NO_3_)_2_], extra pure iron nitrate [Fe(NO_3_)_3_] manganese nitrate [Mn(NO_3_)_2_] and yttrium oxide [Y_2_O_3_] were utilized as initial materials to prepare Mn/Y codoped Sr_1−x_Mn_x_Fe_12−x_Y_x_O_19_ under changing stoichiometric contents (0.0 ≤ x ≤ 0.5) (hereafter designated at MYSNHFs) by sol-gel autocombustion technique. First, stoichiometric amounts of different metal nitrates were dissolved in deionized water using a magnetic stirrer at 80 °C. Next, yttrium oxide was dissolved in 10 mL of HCl at 200 °C by magnetic stirrer to achieve a transparent solution and then added to the nitrate solution under magnetic stirrer at 80 °C for 1 h. Additionally, citric acid (C_6_H_8_O_7_) was added to the resultant mixture as fuel, wherein the pH was adjusted at 7 by incorporating ammonia solution at 150 °C for 30 min, after which the temperature was increased to 320 °C until the solution transformed into a gel then burnt to black powder. Finally, the produced powder was calcinated at 1100 °C for 5 h with heating rate of 10 °C/min to obtain Sr-nHFs phase. 

Structures of as-prepared Sr-nHFs were analyzed using X-ray powder diffraction measurement (XRD; Rigaku Benchtop Miniflex, Tokyo, Japan) operated with Cu Kα line at the angular range of 2θ = 20–70°. Scanning/transmission electron microscope (SEM/TEM; FEI Titan 80 – 300kV FEG S/TEM, Hillsboro, OR, USA) equipped with energy dispersive X-ray (EDX) spectroscopy were used for morphology analysis and to determine the chemical elements present in the sample and elemental mapping. Fourier transform infrared (FTIR; Bruker alpha-II FTIR spectrophotometer attached with a diamond ATR, MA, USA) spectra in the wavenumber range of 4000 to 400 cm^−1^ were recorded to confirm the formation of M-type hexaferrite metal-oxygen bond. AC magnetic susceptibilities and dc magnetizations of all prepared products were measured using a superconducting quantum interference device (PPMS DynaCool, Quantum Design, San Diego, CA, USA). The Mössbauer spectra were performed at room temperature using a conventional Mössbauer spectrometer (Fast Com Tec PC-moss II, Oberhaching, Germany) under constant accelerations mode using ^57^Fe in Rh matrix with an approximate activity of 10 m Ci. The recorded spectra were analyzed and fitted to inbuilt Win-Normos fitting software (WISSEL company, Germany).

## 3. Results and Discussion

### 3.1. Structural Properties

**Figure 1:**
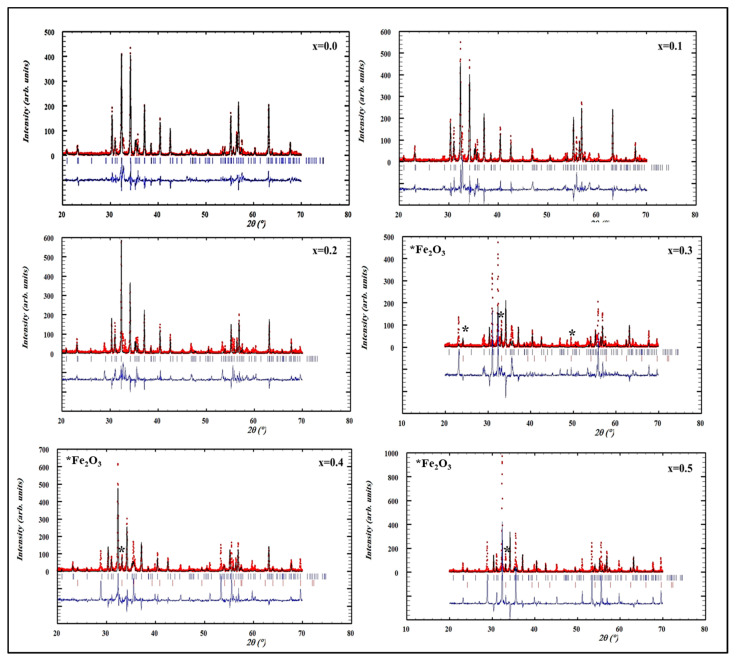
XRD patterns with Rietveld refinement for the various Sr_1−x_Mn_x_Fe_12−x_Y_x_O_19_ (0.0 ≤ x ≤ 0.5) nanohexaferrites.

Figure 1a,b shows the XRD patterns of the studied MYSNHFs, which revealed single hexaferrite phase consistent with the JCPDS Card number 96-100-8857 that implemented through the Rietveld refinements by match3! Software (Crystal Impact, Bonn, Germany). At high dopant (Mn/Y) concentrations, XRD patterns displayed a minor peak assigned to α-Fe_2_O_3_ phase. Rietveld refinements was used to evaluate the cell parameters (a and c) and crystallite size (D) of prepared MYSNHFs as enlisted in Table 1. The value of a was almost reminiscent of the same values with the increase in dopant concentration. However, the observed fluctuation in the *c* values was attributed to the ionic radii mismatch of Fe^3+^ (0.64 Å) and Y^3+^ (0.90 Å) cations that caused a variation in the crystalline microstrain and the exchange energy of the MYSNHFs [30,31]. The crystallite sizes of the obtained MYSNHFs were calculated by Scherrer’s formula wherein full width at half maximum (FWHM) of the most intense XRD peak was selected.

### 3.2. Morphology

**Figure 2:**
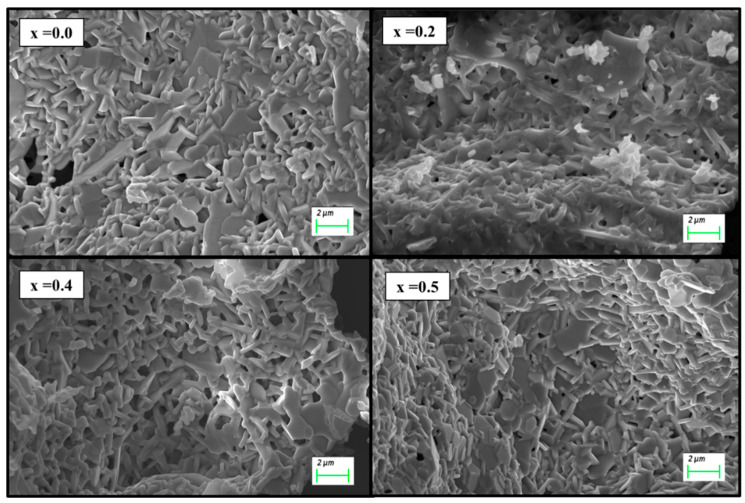
Scanning electron microscope (SEM) images of prepared Sr_1−x_Mn_x_Fe_12−x_Y_x_O_19_ (x = 0.0, 0.2, 0.4, and 0.5) nanohexaferrites.

Figure 2 illustrates the FESEM images of the two selected (x = 0.0, 0.2, 0.4, and 0.5) as-synthesized MYSNHFs, where the surface was consisted of some aggregates of hexagonal plate-like structures. The particles are nanoscale in thickness and microscale in diameter (1–5 µm), so it can be said that the Sr-ferrite particles tend more to grow in the direction parallel to hexagonal plane than that of vertical to the plane [32,33,34].

**Figure 3:**
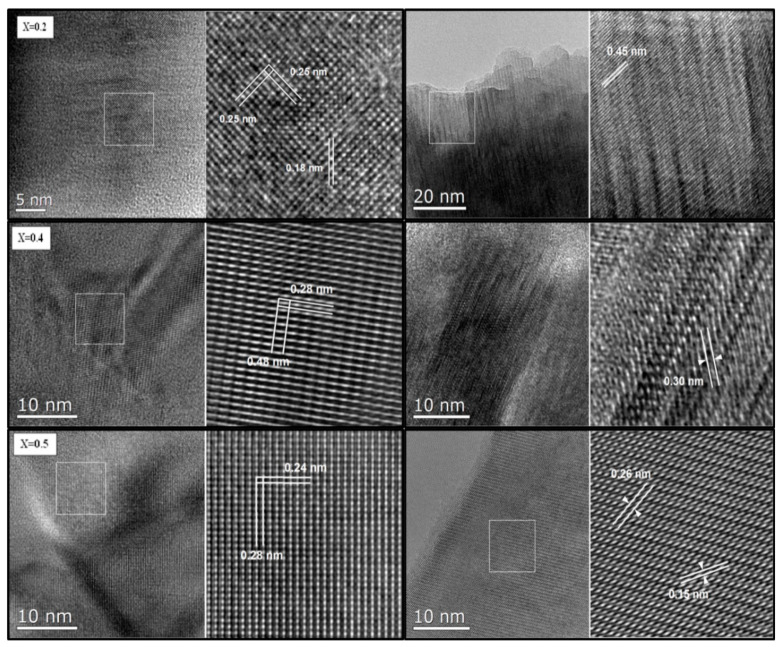
High-resolution transmission electron microscope (HRTEM) images of prepared Sr_1−x_Mn_x_Fe_12−x_Y_x_O_19_ (x = 0.2, 0.4, and 0.5) nanohexaferrites.

Figure 3 depicts the HRTEM images of three selected (x = 0.2, 0.4, and 0.5) MYSNHFs together with their lattice spacing. The values of lattice spacing were found between 0.15 to 0.48 nm. The estimated lattice spacing were tallied to the (307), (209), (203), (108), (114), (008), (106), (102), and (101) orientations of M type hexagonal atomic planes (in accordance to JCPDS card number) for the respective dopant content as indicated in the Figure 3. 

**Figure 4:**
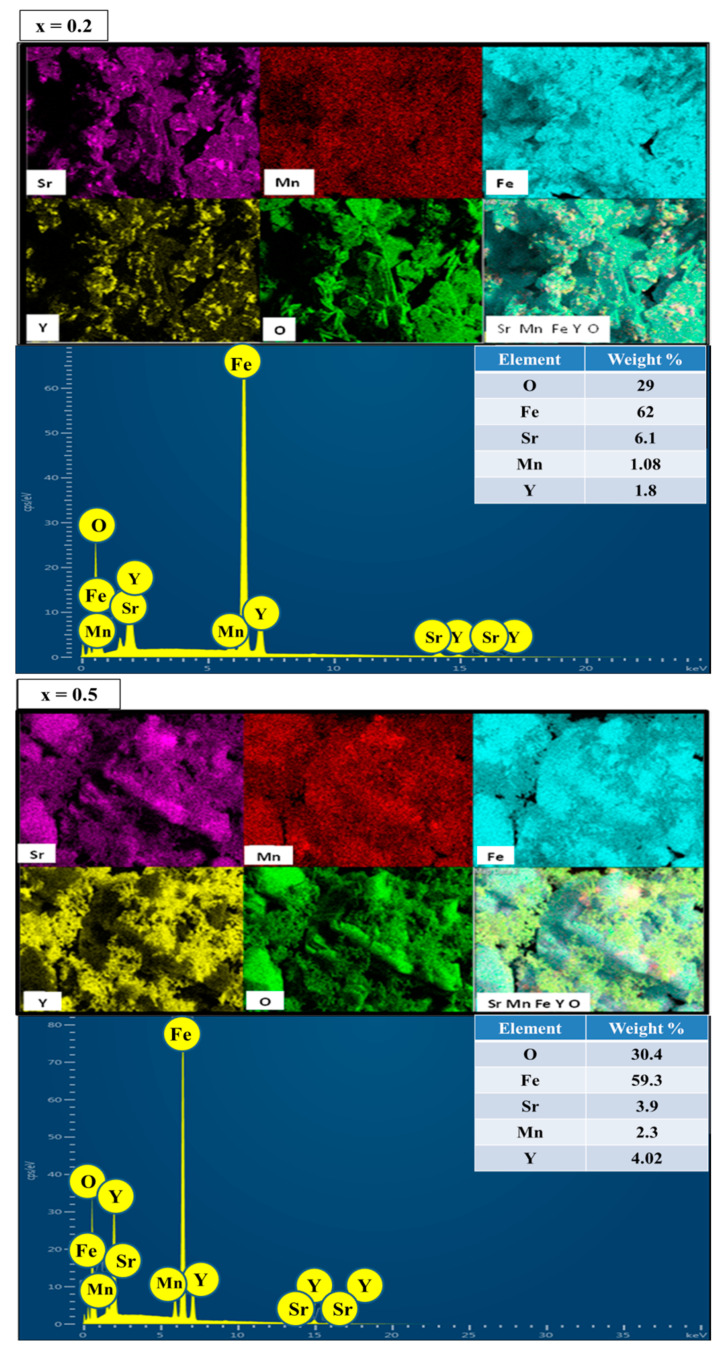
Elemental mapping and energy-dispersive X-ray diffraction spectroscopy (EDX) spectra of Sr_1−x_Mn_x_Fe_12−x_Y_x_O_19_ (x = 0.2 and 0.5) nanohexaferrites.

Figure 4 displays the EDX spectra and elemental maps of two selected (x = y = 0.2 and 0.5) MYSNHFs, which revealed the appropriate traces of elements (correct stoichiometric ratios) as indicated in the inset. This observation clearly confirmed the incorporation of Mn/Y into the Sr–HFs lattice structures.

### 3.3. FTIR Spectra

**Figure 5:**
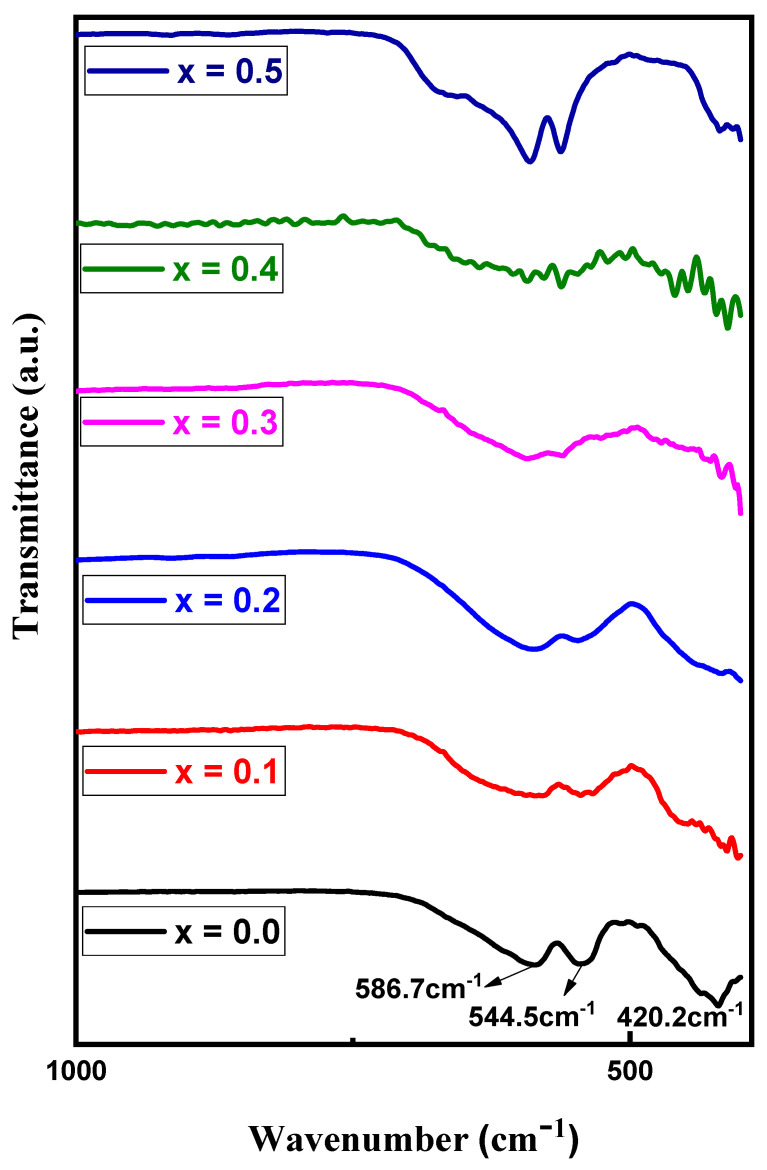
Fourier-transform infrared spectroscopy (FTIR) spectra of proposed Sr_1−x_Mn_x_Fe_12−x_Y_x_O_19_ (0.0 ≤ x = y ≤ 0.5) nanohexaferrites.

Figure 5 presents the FTIR spectra of obtained MYSNHFs, where the spectral features of all samples were nearly the same. The observed absorption bands at ~420.2, ~544.5, and ~586.7 cm^−1^ were assigned to the asymmetric stretching of MYSNHFs linkages and out-of-plane bending vibrations of octahedral as well as tetrahedral sites [35,36]. The appeared bands at around 426.34 and 589.67 cm^−1^ were allocated to the Fe–O bending vibration and Fe–O stretching vibrations. Meanwhile, the observed band at 548.84 cm^−1^ was approved to the Sr–O bending vibration [37]. Besides, all the absorption bands were broadened accompanied by slight shift (so called bands position disorder) with the increase in the Mn^2+^ and Y^3+^ contents in the MYSNHFs [38].

### 3.4. Mössbauer Spectral Analysis

**Figure 6:**
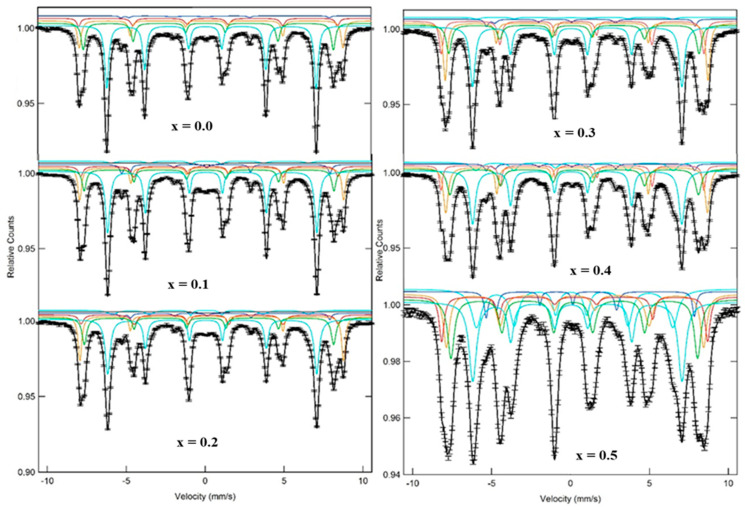
Room temperature Mössbauer spectra of all studied Sr_1−x_Mn_x_Fe_12−x_Y_x_O_19_ (x = 0.0, 0.1, 0.2, 0.3, 0.4 and 0.5) nanohexaferrites.

Figure 6a-f shows the room temperature Mössbauer spectra of achieved MYSNHFs. Mössbauer spectral analyses (using ^57^Fe) were carried out to determine the relationship between the structure and magnetic properties of the proposed MYSNHFs. It provided useful information about the preferred lattice site occupancy of each type of dopant (cations distribution) in the achieved MYSNHFs. 

Table 2 summarizes the fitted parameters of MYSNHFs such as the hyperfine field (B_hf_), the quadrupole shift (QS), the isomer shift (IS), the line width (W), and percentage relative area (R_A_) of the dopant components. Mössbauer spectra were fitted with five discrete sextets corresponding to the octahedral (12k, 4f_2_ and 2a), the tetrahedral (4f_1_), and the trigonal bipyramidal (2b) iron sites. For Fe^3+^ ions, MYSNHFs structure consisted of three spin-up (2a, 2b, and 12k) and two spin-down (4f_1_ and 4f_2_) sublattices [39,40]. The 12k position in the Mössbauer spectra of MYSNHFs was split into 12k and 12k_1_, which were assigned to the perturbation of 12k sites by the presence of Mn^2+^ and Y^3+^ ions in the neighboring sites. One superparamagnetic doublet was created in the codoped sample (for x = y = 0.1 and 0.2) beside the ferromagnetic sextets. For uniform distribution of Fe^3+^ ions, the statistical occupancy corresponding to 12k, 4f_1_, 4f_2_, 2a, and 2b sublattice sites in terms of area must be 50:17:17:8:8 [35]. According to results on relative area of undoped Ba-hexaferrite, 12k, 4f_1_, and 2b positions are close to theoretical values [41]. The 2a position was heavily populated but the 4f_2_ site was less occupied.

Values of isomer shift (I.S) for MYSNHFs provided the information about the nature of chemical bonding of the iron as well as valence state of Fe cations. The values of I.S were in the range of 0.26 to 0.401 mm/s for all sextets and corresponded to the characteristic charge states of Fe^3+^. Furthermore, the isomer shift of 4f_1_ and 2a contributions were increased (Table 2) with the increase in doping levels. The isomer shift of other sites remained unaltered with the addition of dopants. These showed that the s electron density of Fe^3+^ ions at the 4f_1_ and 2a sites decreased but others were not affected by Mn^2+^ and Y^3+^ substitution. 

Values of quadrupole splitting (Q.S) of studied MYSNHFs provided the basic insight about the symmetry of crystal lattice and local distortions. As dopant contents were increased, the Q.S of 4f_1,_ 4f_2_ and 2b sites were slightly reduced, which was attributed to the symmetry perturbation around these sites due to Mn^2+^ and Y^3+^ cation substitution. The room temperature ranking of the hyperfine fields for MYSNHFs containing dopants contents of 0, 0.1, 0.2, 0.3, 0.4, and 0.5 for the five Fe sublattices followed the trend of B_hf_ (12k_1_) < B_hf_ (2b) < B_hf_ (12k) < B_hf_ (4f_1_) < B_hf_ (2a) < B_hf_ (4f_2_) except x, y = 0.4, 0.5. Moreover, the hyperfine fields of 2a site was bigger than that of 4f_2_ site for x, y = 0.4, 0.5. The hyperfine magnetic field (Table 2) of all sites was slightly reduced with the increase in dopants contents. Meanwhile, the hyperfine magnetic field of 2a site was reduced up to x, y = 0.3 and then enhanced. The observed reduction in the hyperfine magnetic field of the proposed MYSNHFs with substitution of dopants was ascribed to nonmagnetic nature of Y^3+^ cations that replaced Fe^3+^ ions in the lattice.

**Figure 7:**
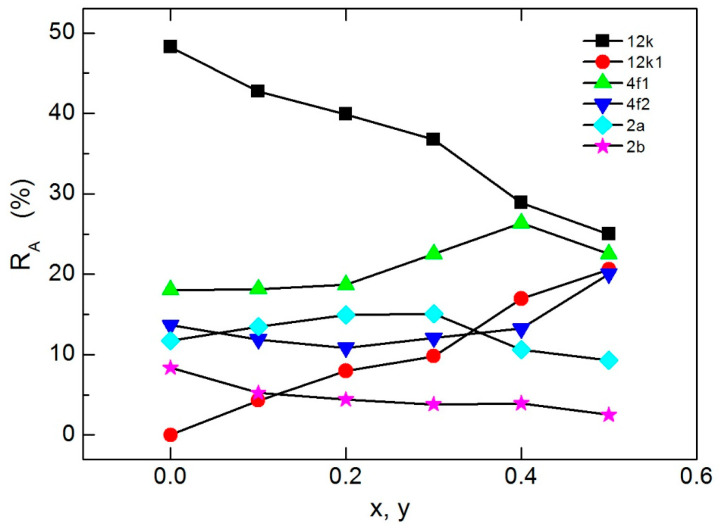
Mn and Y contents dependent relative area variation of Sr_1−x_Mn_x_Fe_12−x_Y_x_O_19_ (0.0 ≤ x ≤ 0.5) nanohexaferrites.

Figure 7 presents the relative area (R_A_) distribution of all sextets for different Mn^2+^/Y^3+^ contents in the obtained MYSNHFs. The value of R_A_ for these sextets was found to be directly proportional to the number of Fe^3+^ cations in the respective site. Besides, the values of R_A_ for 12k, 2b, and 4f_2_ sites were reduced and for the 2a site was increased up to x = 0.3 and further increased thereafter. It was argued that such reduction in R_A_ up to x = 0.3 was due to the preferential occupation of Mn^2+^ and Y^3+^ ions in the 12k, 2b, and 4f_2_ sites. Beyond x = 0.3, the observed increase in the R_A_ value was due to the transfer of some Mn^2+^ and Y^3+^ cations from 4f_2_ site to 2a site. Auwal et al. [42] acknowledged the preferred occupation of Y^3+^ cations at the bipyramidal 2b sites in SrBi_x_La_x_Y_x_Fe_12−3x_O_19_ hexaferrites.

### 3.5. AC Magnetic Susceptibility

**Figure 8:**
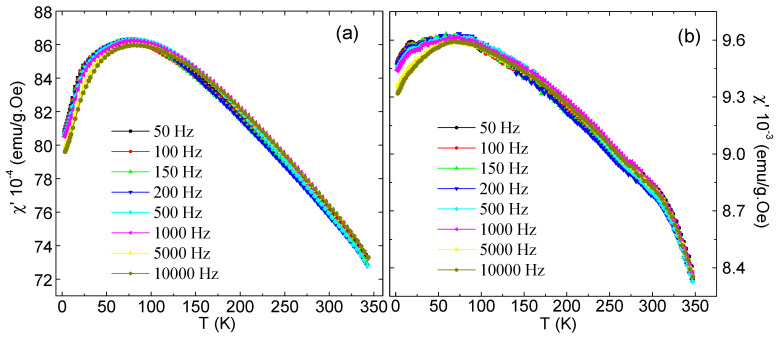
Temperature-dependent AC susceptibility (real part) for (**a**) SrFe_12_O_19_ and (**b**) Sr_0.9_Mn_0.1_Fe_11.9_Y_0.1_O_19_.

**Figure 9:**
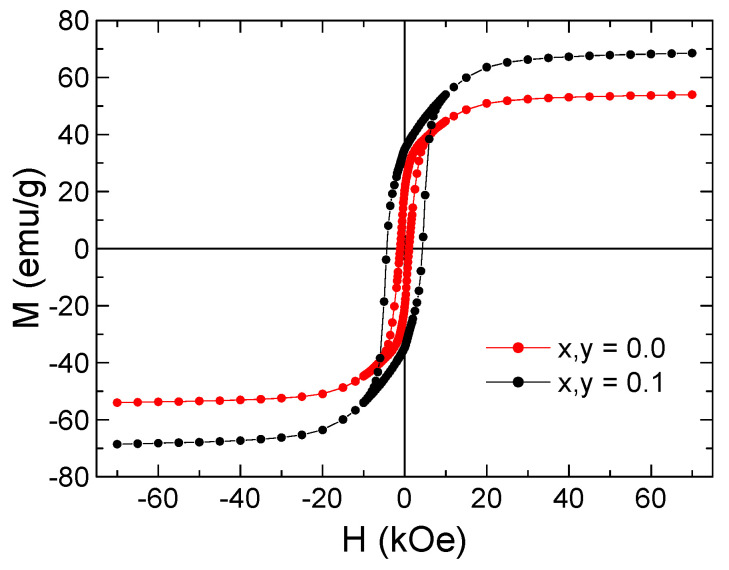
M–H curves of synthesized Sr_1−x_Mn_x_Fe_12−y_Y_y_O_19_ (x = y = 0.0 and 0.1) nanohexaferrites performed at room temperature.

The dynamical magnetic properties and the indirect exchange interactions between Fe^3+^ and Mn^2+^ cations in the synthesized MYSNHFs were evaluated using the AC susceptibility data. Figure 8 illustrates the temperature dependent variation in the real part of the AC-magnetic susceptibility (χ′) for two selected MYSNHFs (with x = 0 and 0.1) subjected to the applied AC-field of 10 Oe over the frequency range of 50 to 10^4^ Hz. The values of χ′ for the Mn^2+^ and Y^3+^ substituted specimens were reduced significantly compared to the undoped (SrFe_12_O_19_) compound, which agreed well with the measurements of magnetization versus applied magnetic field, M(H), as shown in Figure 9. The M(H) hysteresis loops measured at room temperature indicated that the SrFe_12_O_19_ and Sr_0.9_Mn_0.1_Fe_11.9_Y_0.1_O_19_ nanohexaferrites exhibit ferrimagnetic (FM) behavior. It can be clearly seen from the M(H) results that the magnetization is reduced with Mn and Y substitutions. Magnetic parameters including the saturation magnetization, remanence and coercive field were found to decrease with the increase in Mn^2+^ and Y^3+^ contents. Moreover, both samples showed a single peak in χ′(T) curves at a specific temperature T_B_ called blocking temperature, suggesting stabilization of the magnetic phase due to Mn^2+^ and Y^3+^ cation substitutions.

The observed shape of χ′ curve at T_B_ was attributed to the emergence of superparamagnetism (SPM) in MYSNHFs which is shown by numerous spin glass (SG) like states [43,44,45]. In this case, the magnetic moments of the hexaferrite nanoparticles were blocked or frozen at $T<T_{B}$ , otherwise behaved freely like paramagnetic state at $T>T_{B}$ . However, $T_{B}$ is seldom represents the fundamental character of a material but often is determined by the microstructure of the sample. The value of T_B_ for Sr_0.9_Mn_0.1_Fe_11.9_Y_0.1_O_19_ sample (Figure 8) was shifted slightly to higher temperatures compared to the undoped SrFe_12_O_19_ one. This observation was primarily ascribed to the lowering in the anisotropy barrier energy ( $E_{a}$ ) that could determine the SPM state from the blocking region, leading to the grains size shrinkage for x = y = 0.1 [43,44,45]. Additionally, the value of $E_{a}$ was strongly determined by the average particles size and volume ( $E_{a}=K_{\mathrm{eff}}V$ , where $K_{\mathrm{eff}}$ is the effective anisotropy constant and V is the particles volume). The observed shift in T_B_ position towards higher values with the increase in frequency was also reported for numerous spin-frustrated materials [46,47,48].

A small frequency dispersion in χ’ as evidenced on the left-hand side of the freezing peak. In the present study, the behavior of the χ’ clearly indicated the presence of magnetic inhomogeneity of the studied MYSNHFs. Simultaneously, a weak spin relaxation with varying frequency of the external magnetic field occurred. Another evidence of the presence of magnetic inhomogeneity in the prepared MYSNHFs may be the multipeak character of the imaginary part of the ac-susceptibility. However, the absence of significant decrease as well as shift in the peak value of χ’ with the increase in frequency indicated the deficiency of classical spin glass state in the studied MYSNHFs.

**Figure 10:**
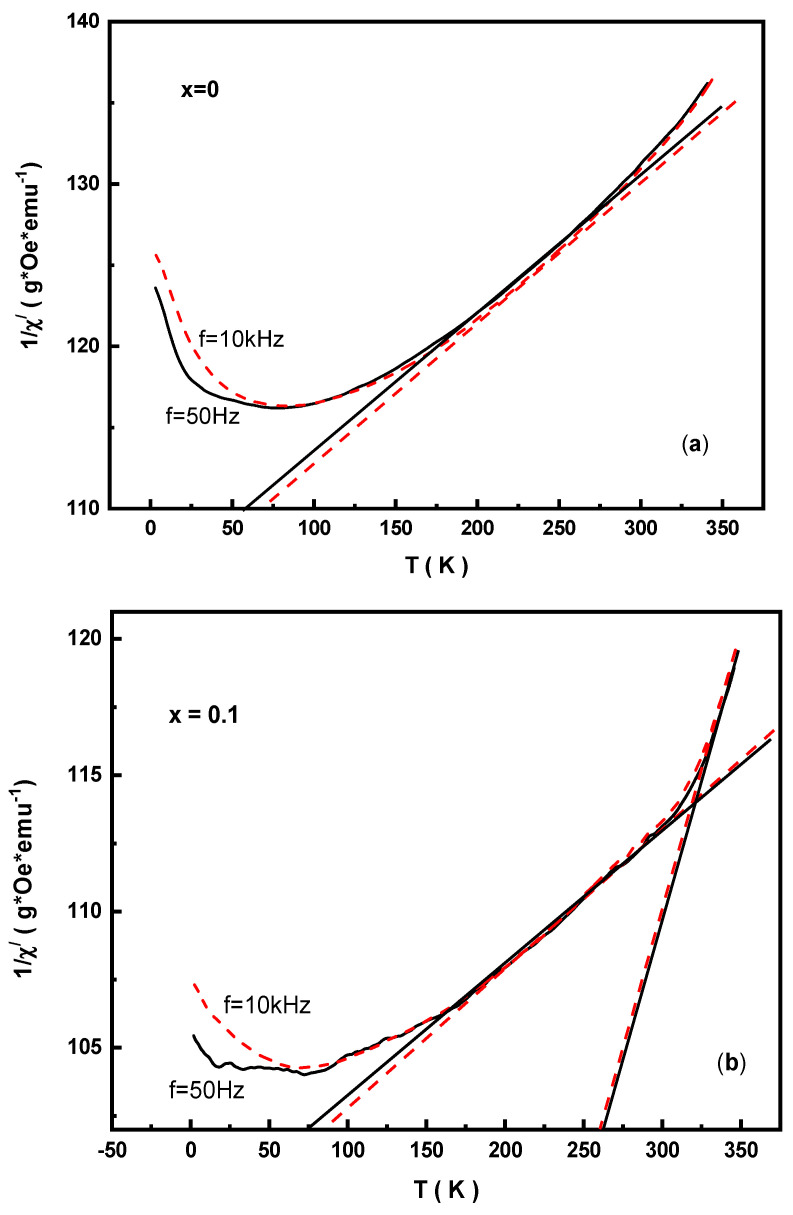
Inverse of real part of the AC susceptibility versus temperature for (**a**) SrFe_12_O_19_ and (**b**) Sr_0.9_Mn_0.1_Fe_11.9_Y_0.1_O_19_.

Figure 10 shows the temperature dependent variation in 1/χ’, wherein some interesting features in the studied MYSNHFs were detected from detailed analysis. The behavior of 1/χ’ versus T was found to be strictly of Curie–Weiss type, in which the paramagnetic Curie temperature (ϴ_p_) was revealed above the Curie point (≈730 K). The attainment of positive ϴ_p_ implied the presence of predominant indirect exchange interactions in the MYSNHFs. In the temperature range of 50 to 350 K, the 1/χ’ curve for SrFe_12_O_19_ was bent downwards, suggesting a continuous change in ϴpi at each point. This in turn indicated the occurrences of a set of positive indirect exchange interactions of different intensities in the studied MYSNHFs.

It is known that in the orbital disordered state, the Mn^2+^(6)–O–Fe^3+^(6) super-exchange interactions for the octahedral coordination of Mn and Fe cations are positive, whereas for the Mn^2+^(5,6)–O–Fe^3+^(6,5) pentahedral coordination they are negative [49,50,51,52]. Thus, due to Mn^2+^ and Y^3+^ cations doping the competitive exchange interactions between the antiferromagnetic and ferromagnetic ordered domains may lead to frustrating exchange coupling and thereby the formation of spin glass state (SGS). The realization of this spin-glass mechanism in the studied MYSNHFs was confirmed by the behavior of 1/χ′ (Figure 10). The linear extrapolation of 1/χ’(*T*) curve above T_B_ provided two different ϴ_p_ values, indicating that the presence of exchange interactions of different strength in the prepared MYSNHFs. Besides, the value of T_B_ also determined the average diameter of the ferromagnetic domains.

The magnetic behavior of particles at nanoscale is known to obey the activation energy of noninteracting magnetic systems likely as in SPM state. Thus, the mechanism of SPM relaxation may be interpreted using the Neel–Arrhenius (N–A) law with the expression [43,44]

(1) $$\tau =\tau_{o}\;\exp\left(E_{a}/k_{B}T_{B}\right)$$

where, τ denotes the measured time related to the applied frequency ( f=1/τ ) and $\tau_{0}$ denotes the jump attempt time of the nanoparticles magnetic moments between opposite orientations (spin flip-flop) of easy axis magnetization, which varies from 10^−9^ to 10^−13^ s for SPM systems. Therefore, the analysis involving the temperature peak shift on the χ′ curves can be regarded as an effective tool to extract the values of $E_{a}$ and $K_{\mathrm{eff}}$ .

**Figure 11:**
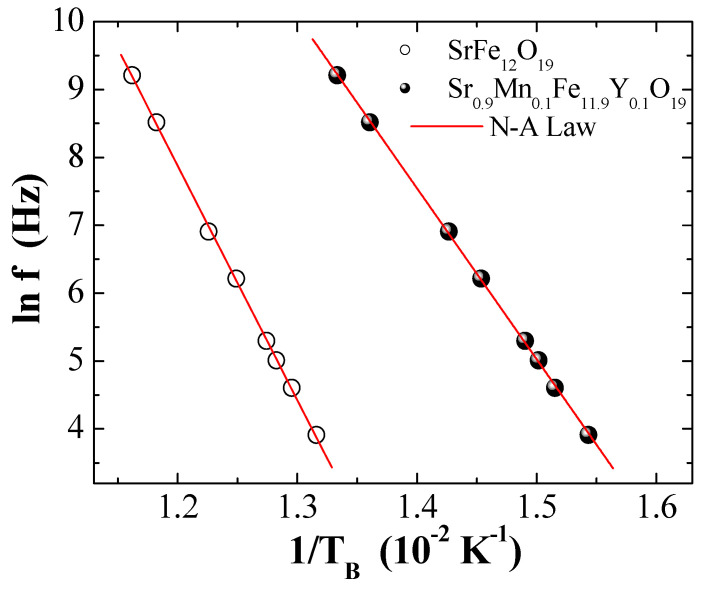
ln(f) against 1/T_B_ for the two selected SrFe_12_O_19_ (i.e., x = y = 0.0) and Sr_0.9_Mn_0.1_Fe_11.9_Y_0.1_O_19_ (i.e., x = y = 0.1) where solid line presents Neel–Arrhenius (N-A) model fit.

Figure 11 illustrates the dependence of ln(f) on 1/T_B_ for two selected MYSNHFs (SrFe_12_O_19_ and Sr_0.9_Mn_0.1_Fe_11.9_Y_0.1_O_19_), wherein the revelation of linear behavior clearly indicated the involvement of thermally activated processes. The slope and the intercept of the ln(f) ~1/T_B_ curves produced the values of $E_{a}$ and $f_{o}=1/\tau_{0}$ , respectively. The values of $K_{eff}$ were obtained from the expression of $E_{a}=K_{\mathrm{eff}}V$ . Table 3 enlists the calculated values of $f_{o}$ , $\tau_{0}$ , $E_{a}/k_{B}$ , and $K_{\mathrm{eff}}$ for the indicated MYSNHFs. In the present work, despite the accurate fitting of the experimental data to the N–A law larger values of $\tau_{o}$ was achieved, which were unphysical and occurred outside the characteristic range shown by SPM systems, signifying the manifestation of strong interaction among MYSNHFs nanoparticles. In short, it was affirmed that N–A theory was deficient to interpret the magnetic traits in these materials.

To get better insight involving the collective response of the magnetic indirect exchange interactions the Vogel–Fulcher (V–F) law was applied [43,44]. In this law, the behavior of the interacting MYSNHFs nanoparticles is given by

(2) $$\tau =\tau_{o}\;\exp\left[E_{a}/k_{B}(T_{B}-T_{0}\right)]$$

where $T_{0}$ denotes the V–F temperature that renders useful information related to the interaction’s intensity between magnetic nanoparticles, $k_{B}$ is the Boltzmann constant, and the other parameters have their usual meaning ( $\tau_{o}$ ≈ 10^−9^ − 10^−13^ s).

**Figure 12:**
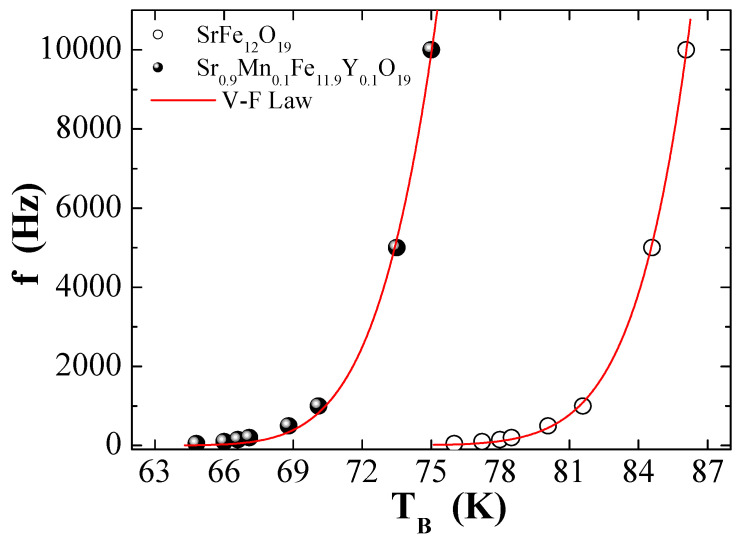
Frequency dependent T_B_ for two selected Sr_1−x_Mn_x_Fe_12−y_Y_y_O_19_ (x = y = 0.0 and 0.1) nanohexaferrites where solid line presents Vogel–Fulcher (V–F) law fit.

Figure 12 presents the dependence of f on T_B_ for two selected MYSNHFs (SrFe_12_O_19_ and Sr_0.9_Mn_0.1_Fe_11.9_Y_0.1_O_19_) together with the V–F law fitting. Table 3 outlines the calculated magnetic parameters, such as the $f_{o}$ , $\tau_{0}$ , $E_{a}/k_{B}$ , and $K_{\mathrm{eff}}$ for the indiacted MYSNHFs. The disclosure of somewhat realistic $\tau_{0}$ values in the allowed range of 10^−9^–10^−13^ s clearly approved the validity of V–F law to describe the achieved magnetic behavior of MYSNHFs in a better way compared to the N–A law. Furthermore, the attainment of non-negligible T_0_ values compared to T_B_ was majorly ascribed to the strong interactions among MYSNHFs nanoparticles in the studied hexaferrites [37]. Meanwhile, the observed shortening in the $\tau_{0}$ values with the inclusion of Mn^2+^ and Y^3+^ cations in the MYSNHFs was mainly attributed to the shrinkage of magnetic nanoparticles and subsequent reduction in the exchange coupling strength among tiny nanoparticles. The values of $K_{\mathrm{eff}}$ and $T_{0}$ for Sr_0.9_Mn_0.1_Fe_11.9_Y_0.1_O_19_ significantly decreased compared to the undoped MYSNHFs (SrFe_12_O_19_). The observed reduction in the $K_{\mathrm{eff}}$ and $T_{0}$ values due to Mn^2+^ and Y^3+^ cations substitution was attributed to the weakening in the magnetic anisotropy or indirect exchange interactions between MYSNHFs nanoparticles [43,44].

## 4. Conclusions

A series of MYSNHFs with stoichiometric composition of Sr_1−x_Mn_x_Fe_12−x_Y_x_O_19_ (0.0 ≤ x ≤ 0.5) were prepared using the citrate sol-gel autocombustion technique. The influence of Mn^2+^ and Y^3+^ ions substitution on the evolution of morphology, microstructure, and magnetic properties of synthesized MYSNHFs was examined. As-prepared samples were characterized using XRD, FESEM, EDX, HRTEM, FTIR, ^57^Fe Mössbauer spectroscopy, and PPMS-VSM measurements. The XRD pattern, FESEM, and HRTEM images confirmed the evolution of M-type hexagonal phases in the achieved MYSNHFs. Mössbauer spectral analyses revealed the preferred occupation of the substitution ions into 12k, 4f_2_, and 2b sites of the hexagonal sublattice in MYSNHFs. The AC susceptibility (real part) of the proposed MYSNHFs disclosed strong frequency dependent magnetic response. The observed shift in the AC susceptibility peak towards lower T_B_ value with increasing Mn^2+^and Y^3+^ ions substitution levels was attributed to the shrinkage of magnetic nanoparticles in the studied MYSNHFs. It was established that the magnetic interactions were weakened due to the inclusion of Mn^2+^and Y^3+^ ions into prepared MYSNHFs, wherein Sr^2+^ and Fe^3+^ ions were replaced by respective Mn^2+^ and Y^3+^ ions. In short, present knowledge may contribute towards the development of Mn^2+^ and Y^3+^ ions substituted MYSNHF-based device applications.

## Figures and Tables

**Table 1:** Structural parameters of all the synthesized Sr_1−x_Mn_x_Fe_12−x_Y_x_O_19_ (0.0 ≤ x ≤ 0.5) nanohexaferrites.

x	a = b (Å)	c (Å)	D_XRD_ (nm)	χ^2^
0.0	5.881	23.048	55.1	1.8
0.1	5.881	23.021	69.1	2.1
0.2	5.882	23.023	55.9	2.4
0.3	5.883	23.039	59.8	2.6
0.4	5.883	23.029	49.5	2.6
0.5	5.884	23.050	37.4	3.2

**Table 2:** Mössbauer spectral parameters of the studied Sr_1−x_Mn_x_Fe_12−x_Y_x_O_19_ (0.0 ≤ x ≤ 0.5) nanohexaferrites including hyperfine magnetic field (B_hf_), isomer shift (I.S), quadrupole splitting (Q.S), line width (W), and relative area percent (R_A_%) with estimated uncertainties.

x	Site	B_hf_ (T)	I.S (mm/s)	Q.S (mm/s)	W (mm/s)	R_A_ (%)
		(±0.01)	(±0.002)	(±0.001)	(±0.006)	
**0**	12k	41.183	0.353	0.396	0.277	48.381
	4f_1_	49.157	0.259	0.176	0.238	17.771
	4f_2_	51.835	0.379	0.292	0.244	13.924
	2a	50.885	0.323	0.016	0.363	11.679
	2b	40.937	0.279	2.279	0.988	8.2449
**0.1**	12k	41.13	0.353	0.401	0.249	43.331
	12k_1_	38.761	0.305	0.223	0.348	4.1509
	4f_1_	49.106	0.261	0.179	0.248	16.983
	4f_2_	51.896	0.375	0.285	0.254	12.616
	2a	50.558	0.342	0.069	0.311	13.347
	2b	40.952	0.292	2.247	0.251	5.3067
	Db	-	0.232	0.703	680	4.2661
**0.2**	12k	41.089	0.351	0.399	0.251	39.926
	12k_1_	38.557	0.305	0.271	0.266	7.995
	4f1	48.994	0.261	0.171	0.267	18.46
	4f2	51.814	0.362	0.317	0.146	10.953
	2a	50.464	0.356	0.058	0.313	15.042
	2b	40.879	0.305	2.216	0.264	4.3767
	Db	-	0.293	0.871	0.814	3.2476
**0.3**	12k	41.154	0.354	0.4	0.294	33.051
	12k_1_	38.652	0.284	0.222	0.234	9.0202
	4f_1_	49.08	0.279	0.127	0.343	19.982
	4f_2_	52.551	0.422	−0.041	0.2	8.7147
	2a	50.885	0.384	0.015	0.449	23.917
	2b	40.983	0.297	2.244	0.268	3.5987
	Db	-	0.438	0.835	0.812	1.7174
**0.4**	12k	41.113	0.346	0.389	0.302	28.237
	12k_1_	38.762	0.27	0.217	0.304	16.523
	4f_1_	48.732	0.292	0.111	0.402	21.383
	4f_2_	52.555	0.423	−0.078	0.18	5.1586
	2a	50.816	0.373	0.089	0.471	24.509
	2b	40.906	0.282	2.187	0.249	3.2939
	Db	-	0.249	0.39	0.92	0.89625
**0.5**	12k	41.103	0.35	0.385	0.463	25.222
	12k_1_	38.589	0.258	0.225	0.461	20.37
	4f_1_	48.464	0.315	0.063	0.511	22.301
	4f_2_	52.221	0.403	−0.07	0.467	10.131
	2a	50.509	0.376	0.04	0.547	19.531
	2b	40.914	0.284	2.14	0.21	2.4454

**Table 3:** Physical parameters of the selected Sr_1−x_Mn_x_Fe_12−y_Y_y_O_19_ (x = y = 0.0 and 0.1) nanohexaferrites obtained from Neel–Arrhenius and Vogel–Fulcher model fitting.

Models	Parameters	Values	
		SrFe_12_O_19_	Sr_0.9_Mn_0.1_Fe_11.9_Y_0.1_O_19_
Neel–Arrhenius	$\tau_{o}$ (s)	3.85 × 10^−22^	2.58 × 10^−19^
	$E_{a}/k_{B}$ (K)	3452.59	2518.3
	$K_{\mathrm{eff}}$ (erg/cm^3^)	1.25 × 10^3^	2.02 × 10^3^
Vogel–Fulcher	$\tau_{o}$ (s)	1.18 × 10^−11^	7.39 × 10^−11^
	$E_{a}/k_{B}$ (K)	587.83	226.66
	$T_{o}$ (K)	49.22	51.26
	$K_{\mathrm{eff}}$ (erg/cm^3^)	213.33	181.94

## References

[1] Rao C.N.R., Cheetham A.K. (2001). Science and technology of nanomaterials: Current status and future prospects. J. Mat. Chem..

[2] Wang X.X., Ma T., Shu J.C., Cao M.S. (2018). Confinedly tailoring Fe_3_O_4_ clusters-NG to tune electromagnetic parameters and Microwave absorption with broadened bandwidth. Chem. Eng. J..

[3] Gu Z., Yan L., Tian G., Li S., Chai Z., Zhao Y. (2013). Recent Advances in Design and Fabrication of Upconversion Nanoparticles and Their Safe Theranostic Applications. Adv. Mater..

[4] Katlakunta S., Meena S.S., Srinath S., Bououdina M., Sandhya R., Praveena K. (2015). Improved magnetic properties of Cr^3+^ doped SrFe_12_O_19_ synthesized via microwave hydrothermal route. Mater. Res. Bull..

[5] Jacobo S.E., Bercoff P.G., Herme C.A., Vives L.A. (2015). Sr hexaferrite/Ni ferrite nanocomposites magnetic behavior and microwave absorbing properties in the X-band. Mater. Chem. Phys..

[6] Auwal I.A., Baykal A., Güner S., Sertkol M., Sözeri H. (2016). Magneto-optical properties BaBi_x_La_x_Fe_12−2x_O_19_ (0.0 ≤ x ≤ 0.5) hexaferrites. J. Magn. Magn. Mater..

[7] Harker S., Stewart G., Hutchison W., Amiet A., Tucker D. (2015). Microwave absorption and ^57^Fe Mössbauer properties of Ni-Ti doped barium hexaferrite. Hyperfine Interact..

[8] Dishovski N., Petkov A., Nedkov I., Razkazov I. (1994). Hexaferrite contribution to microwave absorbers characteristics. IEEE Trans. Magn..

[9] Iqbal M.J., Ashiq M.N., Gomez P.H., Munoz J.M. (2008). Synthesis, physical, magnetic and electrical properties of Al–Ga substituted co-precipitated nanocrystalline strontium hexaferrite. J. Magn. Magn. Mater..

[10] Obradors X., Collomb A., Pernet M., Joubert J.C., Isalgue A. (1984). Structural and magnetic properties of BaFe_12−x_Mn_x_O_19_ hexagonal ferrites. J. Magn. Magn. Mater..

[11] Mocuta H., Lechevallier L., le Breton J.M., Wang J.F., Harris I.R. (2004). Structural and magnetic properties of hydrothermally synthesized Sr_1−*x*_Nd*_x_*Fe_12_O_19_ hexagonal ferrites. J. Alloy. Compd..

[12] Silva W.M.S., Ferreira N.S., Soares J.M., da Silva R.B., Macêdo M.A. (2015). Investigation of structural and magnetic properties of nanocrystalline Mn-doped SrFe_12_O_19_ prepared by proteic sol–gel process. J. Magn. Magn. Mater..

[13] Jiang S., Liu X., Rehman K.M.U., Li M., Wu Y. (2016). Synthesis and characterization of Sr_1−x_Y_x_Fe_12_O_19_ hexaferrites prepared by solid-state reaction method. J. Mater. Sci. Mater. Electron..

[14] Niu X.F., Zhang M.Y. (2014). Structure and magnetic properties of yttrium-doped M-type strontium ferrite. Asian J. Chem..

[15] Shekhawat D., Roy P.K. (2018). Impact of yttrium on the physical, electro-magnetic and dielectric properties of auto-combustion synthesized nanocrystalline strontium hexaferrite. J. Mater. Sci. Mater. Electron..

[16] Almessiere M.A., Slimani Y., el Sayed H.S., Baykal A. (2018). Structural and magnetic properties of Ce-Y substituted strontium nanohexaferrites. Ceram. Int..

[17] Almessiere M.A., Slimani Y., Baykal A. (2019). Impact of Nd-Zn co-substitution on microstructure and magnetic properties of SrFe_12_O_19_ nanohexaferrite. Ceram. Int..

[18] Peng L., Li L.Z., Wang R., Hu Y., Tu X.Q., Zhong X.X. (2015). Effect of La–Co substitution on the crystal structure and magnetic properties of low temperature sintered Sr_1−x_La_x_Fe_12−x_Co_x_O_19_ (x=0–0.5) ferrites. J. Magn. Magn. Mater..

[19] Yang Y.J., Liu X.S. (2014). Microstructure and magnetic properties of La– Cu doped M-type strontium ferrites prepared by ceramic process. Mater. Technol. Adv. Perform. Mater..

[20] Yang Y., Wang F., Shao J., Batoo K.M. (2017). Microstructure and magnetic properties of Zr–Mn substituted M-type SrLa hexaferrites. Appl. Phys. A.

[21] Zhang Z.Y., Liu X.X., Wang X.J., Peng Y., Li R. (2012). Effect of Nd–Co substitution on magnetic and microwave absorption properties of SrFe12O19 hexaferrites. J. Alloy. Compd..

[22] Lee S.W., An S.Y. (2005). In-Bo Shim, Chul Sung Kim, Mossbauer studies of La–Zn substitution effect in strontium ferrite nanoparticles. J. Magn. Magn. Mater..

[23] Yang Y., Wang F., Shao J., Huang D., He H., Trukhanov A.V., Trukhanov S.V. (2018). Influence of Nd-NbZn co-substitution on structural, spectral and magnetic properties of M-type calcium-strontium hexaferrites Ca_0.4_Sr_0.6-x_Nd_x_Fe_12.0-x_(Nb_0.5_Zn_0.5_)_x_O_19_. J. Alloy. Compd..

[24] Ashiq M.N., Shakoor S., Najam-ul-Haq M., Warsi M.F., Ali I., Shakir I. (2015). Structural, electrical, dielectric and magnetic properties of Gd-Sn substituted Sr-hexaferrite synthesized by sol–gel combustion method. J. Magn. Magn. Mater..

[25] Iqbal M.J., Farooq S. (2010). Impact of Pr–Ni substitution on the electrical and magnetic properties of chemically derived nanosized strontium–barium hexaferrites. J. Alloy. Compd..

[26] Shakoor S., Ashiq M.N., Marlana M.A., Mahmood A., Warsi M.F., Najam-ul-Haq M., Karamat N. (2014). Electrical, dielectric and magnetic characterization of Bi–Cr substituted M-type strontium hexaferrite nanomaterials. J. Magn. Magn. Mater..

[27] Kaur P., Chawla S.K., Meena S.S., Yusuf S.M., Pubby K., Narang S.B. (2017). Modulation of physico-chemical, magnetic, microwave and electromagnetic properties of nanocrystalline strontium hexaferrite by Co-Zr doping synthesized using citrate precursor sol-gel method. Ceram. Int..

[28] Joshi R., Singh C., Kaur D., Zaki H., Narang S.B., Jotania R., Mishra S., Singh J., Dhruv P., Ghimiree M. (2017). Structural and magnetic properties of Co^2+^-W^4+^ ions doped M-type Ba-Sr hexaferrites synthesized by a ceramic method. J. Alloy. Compd..

[29] Yang Y.J., Shao J.X., Wang F.H., Liu X.S., Huang D.H. (2017). Impacts of MnZn doping on the structural and magnetic properties of M-type SrCaLa hexaferrites. Appl. Phys. A.

[30] Baniasadi A., Ghasemi A., Nemati A., Ghadikolaei M.A., Paimozd E. (2014). Effect of Ti–Zn substitution on structural, magnetic and microwave absorption characteristics of strontium hexaferrite. J. Alloy. Compd..

[31] Rostami M., Vahdani M.R.K., Moradi M., Mardani R. (2017). Structural, magnetic, and microwave absorption properties of Mg–Ti–Zr–Co-substituted barium hexaferrites nanoparticles synthesized via sol–gel auto-combustion method. J. Sol-Gel Sci. Technol..

[32] Zhang T., Peng X., Li J., Yang Y., Xu J., Wang P., Jin D., Jin H., Hong B., Wang X. (2016). Platelet-like hexagonal SrFe_12_O_19_ particles: Hydrothermal synthesis and their orientation in a magnetic field. J. Magn. Magn. Mater..

[33] Rezaie E., Rezanezhad A., Ghadimi L.S., Hajalilou A., Arsalani N. (2018). Effect of calcination on structural and supercapacitance properties of hydrothermally synthesized plate-like SrFe_12_O_19_ hexaferrite nanoparticles. Ceram. Int..

[34] Annapureddya V., Kang J.H., Palneedi H., Kima J.W., Ahna C.W., Choi S.Y., Johnson S.D., Ryu J. (2017). Growth of self-textured barium hexaferrite ceramics by normal sintering process and their anisotropic magnetic properties. J. Eur. Ceram. Soc..

[35] Pradeep A., Chandrasekaran G. (2006). FTIR study of Ni, Cu and Zn substituted nano-particles of MgFe_2_O_4_. Mater. Lett..

[36] Pereira F.M.M., Junior C.A.R., Santos M.R.P., Sohn R.S.T.M., Freire F.N.A., Sasaki J.M., De-Paiva J.A.C., Sombra A.S.B. (2008). Structural and dielectric spectroscopy studies of the M-type barium strontium hexaferrite alloys (Ba*_x_*Sr_1−*x*_Fe_12_O_19_). J. Mater. Sci. Mater. Electron..

[37] Thakur A., Singh R.R., Barman P.B. (2013). Synthesis and characterizations of Nd^3+^ doped SrFe_12_O_19_ nanoparticles. Mater. Chem. Phys..

[38] Tenorio-Gonzalez F.N., Bolarín-Miro A.M., Jesús F.Sa., Vera-Serna P., Menendez-Gonzalez N., Sanchez-Marcos J. (2017). Crystal structure and magnetic properties of high Mn-doped strontium hexaferrite. J. Alloy. Compd..

[39] Solovyova E.D., Pashkova E.V., Ivanitski V.P., Vyunov O.I., Belous A.G. (2013). Mössbauer and X-ray diffraction study of Co^2+^–Si^4+^ substituted M-type barium hexaferrite BaFe_12−2*x*_Co*_x_*Si*_x_*O_19±*γ*_. J. Magn. Magn. Mater..

[40] Belous A.G., Vyunov O.I., Pashkova E.V., Ivanitski V.P., Gavrilenko O.N. (2006). Mössbauer Study and Magnetic Properties of M-Type Barium Hexaferrite Doped with Co + Ti and Bi + Ti Ions. Phys. Chem. B.

[41] Chawla S.K., Mudsainiyan R.K., Meena S.S., Yusuf S.M. (2014). Sol–gel synthesis, structural and magnetic properties of nanoscale M-type barium hexaferrites BaCo*_x_*Zr*_x_*Fe_(12−2*x*)_O_19_. J. Magn. Magn. Mater..

[42] Auwal I.A., Güngüneş H., Güner S., Shirsath S.E., Sertkol M., Structural A.B. (2016). magneto-optical properties and cation distribution of SrBi_x_La_x_Y_x_Fe_12−3x_O_19_ (0.0 ≤ x ≤ 0.33) hexaferrites. Mater. Res. Bull..

[43] Almessiere M.A., Slimani Y., Güngüneş H., el Sayed H.S., Baykal A. (2018). AC susceptibility and Mossbauer study of Ce^3+^ ion substituted SrFe_12_O_19_ nanohexaferrites. Ceram. Int..

[44] Almessiere M.A., Slimani Y., el Sayed H.S., Baykal A. (2018). Ce-Y co-substituted Strontium nanohexaferrites: AC susceptibility and Mossbauer studies. Ceram. Int..

[45] Slimani Y., Baykal A., Manikandan A. (2018). Effect of Cr^3+^ substitution on AC susceptibility of Ba hexaferrite nanoparticles. J. Magn. Magn. Mater..

[46] Mohapatra J., Mitra A., Bahadur D., Aslam M. (2015). Superspin glass behavior of self-interacting CoFe_2_O_4_ nanoparticles. J. Alloy. Compd..

[47] Kaul S.N., Methfessel S. (1983). Effect of field and frequency on the temperature dependence of a.c. susceptibility of the (La, Gd) Ag spin-glass. Solid State Comm..

[48] Fiorani D., Viticoli S., Dormann J.L., Tholence J.L., Murani A.P. (1984). Spin-glass behavior in an antiferromagnetic frustrated spinel: ZnCr_1.6_Ga_0.4_O_4_. Phys. Rev. B.

[49] Trukhanov S.V., Troyanchuk I.O., Fita I.M., Szymczak H., Bärner K. (2001). Comparative study of the magnetic and electrical properties of Pr_1−x_Ba_x_MnO_3-δ_ manganites depending on the preparation conditions. J. Magn. Magn. Mater..

[50] Trukhanov S.V., Lobanovski L.S., Bushinsky M.V., Khomchenko V.A., Pushkarev N.V., Tyoyanchuk I.O., Maignan A., Flahaut D., Szymczak H., Szymczak R. (2004). Influence of oxygen vacancies on the magnetic and electrical properties of La_1−x_Sr_x_MnO_3−x/2_ manganites. Eur. Phys. J. B.

[51] Trukhanov S.V., Trukhanov A.V., Vasiliev A.N., Szymczak H. (2010). Frustrated exchange interactions formation at low temperatures and high hydrostatic pressures in La_0.70_Sr_0.30_MnO_2.85_. J. Exp. Theor. Phys..

[52] Trukhanov S.V., Trukhanov A.V., Turchenko V.A., Kostishyn V.G., Panina L.V., Kazakevich I.S., Balagurov A.M. (2016). Structure and magnetic properties of BaFe_11.9_In_0.1_O_19_ hexaferrite in a wide temperature range. J. Alloy. Compd..

