# Correction: Almessiere et al. Manganese/Yttrium Codoped Strontium Nanohexaferrites: Evaluation of Magnetic Susceptibility and Mossbauer Spectra. *Nanomaterials* 2019, *9*, 24

**DOI:** 10.3390/nano15191464

**Published:** 2025-09-24

**Authors:** Munirah Abdullah Almessiere, Yassine Slimani, Hakan Güngüneş, Abdulhadi Baykal, S.V. Trukhanov, A.V. Trukhanov

**Affiliations:** 1 Department of Physics, College of Science, Imam Abdulrahman Bin Faisal University, P.O. Box 1982, Dammam 31441, Saudi Arabia; 2 Department of Nano-Medicine Research, Institute for Research & Medical Consultations (IRMC), Imam Abdulrahman Bin Faisal University, P.O. Box 1982, Dammam 31441, Saudi Arabia; abaykal@iau.edu.sa; 3 Department of Biophysics, Institute for Research & Medical Consultations (IRMC), Imam Abdulrahman Bin Faisal University, P.O. Box 1982, Dammam 31441, Saudi Arabia; yaslimani@iau.edu.sa; 4 Department of Physics, Hitit University, Çevre Yolu Bulvarı-Çorum 19030, Turkey; Gungunes@gmail.com; 5 Scientific-Practical Materials Research Centre NAS of Belarus, 19 P. Brovki Street, 220072 Minsk, Belarus; trukhanov@gmail.com (S.V.T.); truhanov86@mail.ru (A.V.T.); 6 Department of Electronic Materials Technology, National University of Science and Technology MISiS, Leninsky Prospekt, 4, Moscow 119049, Russia; 7 Laboratory of Crystal Growth, South Ural State University, Lenin Prospect, 76, Chelyabinsk 454080, Russia

In the original publication [1], there was a mistake in Figure 2 as published. The corrected Figure 2 appears below. The authors state that the scientific conclusions are unaffected. This correction was approved by the Academic Editor. The original publication has also been updated.

## Figures and Tables

**Figure 2:**
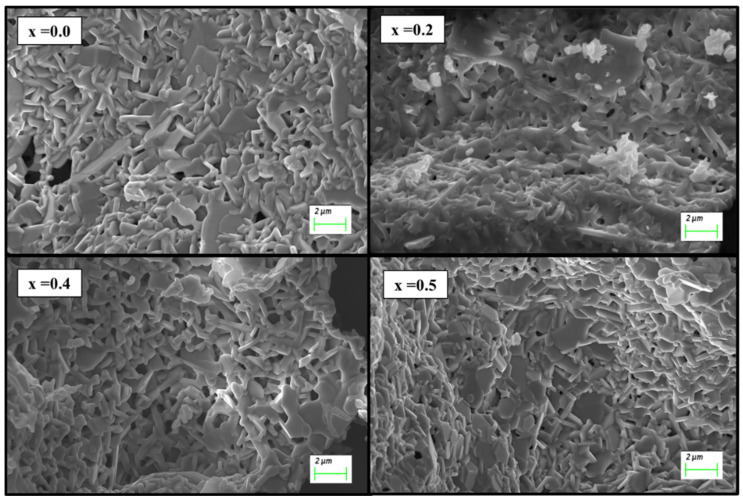
Scanning electron microscope (SEM) images of prepared Sr_1−x_Mn_x_Fe_12−x_Y_x_O_19_ (x = 0.0, 0.2, 0.4, and 0.5) nanohexaferrites.
